# Prefrontal fNIRS-based clinical data analysis of brain functions in individuals abusing different types of drugs

**DOI:** 10.1186/s13326-021-00256-y

**Published:** 2021-11-25

**Authors:** Xuelin Gu, Banghua Yang, Shouwei Gao, Lin Feng Yan, Ding Xu, Wen Wang

**Affiliations:** 1 School of Mechanical and Electrical Engineering and Automation, Shanghai University, Shanghai, 200444 China; 2 Department of Radiology & Functional and Molecular Imaging Key Lab of Shaanxi Province, Tangdu Hospital, Fourth Military Medical University, Xi’an, 710038 Shaanxi China; 3 Shanghai Drug Rehabilitation Administration Bureau, Shanghai, 200080 China

**Keywords:** Drug addiction, fNIRS, Clinical data analysis, Classification of different drug users, OFC activation

## Abstract

**Background:**

The activation degree of the orbitofrontal cortex (OFC) functional area in drug abusers is directly related to the craving for drugs and the tolerance to punishment. Currently, among the clinical research on drug rehabilitation, there has been little analysis of the OFC activation in individuals abusing different types of drugs, including heroin, methamphetamine, and mixed drugs. Therefore, it becomes urgently necessary to clinically investigate the abuse of different drugs, so as to explore the effects of different types of drugs on the human brain.

**Methods:**

Based on prefrontal high-density functional near-infrared spectroscopy (fNIRS), this research designs an experiment that includes resting and drug addiction induction. Hemoglobin concentrations of 30 drug users (10 on methamphetamine, 10 on heroin, and 10 on mixed drugs) were collected using fNIRS and analyzed by combining algorithm and statistics.

**Results:**

Linear discriminant analysis (LDA), Support vector machine (SVM) and Machine-learning algorithm was implemented to classify different drug abusers. Oxygenated hemoglobin (HbO2) activations in the OFC of different drug abusers were statistically analyzed, and the differences were confirmed. Innovative findings: in both the Right-OFC and Left-OFC areas, methamphetamine abusers had the highest degree of OFC activation, followed by those abusing mixed drugs, and heroin abusers had the lowest. The same result was obtained when OFC activation was investigated without distinguishing the left and right hemispheres.

**Conclusions:**

The findings confirmed the significant differences among different drug abusers and the patterns of OFC activations, providing a theoretical basis for personalized clinical treatment of drug rehabilitation in the future.

## Background

Clinically, there has been little theoretical support for the effects of brain functions abusing different types of drugs such as heroin, methamphetamine, and mixed drugs. Moreover, the high degree of OFC activations in drug users indicates a high degree of cravings for drugs and a high tolerance to punishment.

The paper mainly highlights the following six points: Forehead fNIRS is first applied to drug addiction clinics; Designing a special experimental paradigm process to collect clinical data of people taking different types of addictive drugs; Traditional evaluation of drug abuse types uses statistical scales and addicts’ account, which is subjective. This article proposes using LDA, SVM and CNN algorithms to classify people taking different drugs, and objectively judge the types of drug abuse, overcoming the shortcoming of traditional evaluation mode of being subjective; Statistics is used to analyze the differences of OFC functional area activation; Warning people on social impacts, informing them of the harmfulness of drugs and necessity to refuse drugs; Clinically, providing a theoretical basis for doctors in differentiated rehabilitation treatment.

fNIRS is a new research field, and its advantages are received by researchers. Optical brain imaging functional near infrared spectroscopy [1, 2] is a spectral measurement based on scalp detection. It measures the hemodynamic function of brain tissues based on optic injection and detection points, and records blood oxygen level [3]. Compared with the emerging brain function imaging modality of EEG, NIRS is easy to wear, resistant to interference, and portable [4]. The oxyhemoglobin (HbO2) and deoxyhemoglobin (Hbb) in human body have specific absorption for the near-infrared light with a wavelength of 600 nm to 900 nm, while other biological tissues in the brain are relatively transparent in this range of wavelength. Therefore, NIRS is an ideal choice to measure the changes in the intensity of near-infrared light with a wavelength of 600 nm to 900 nm injected into the brain tissue, while the indirect brain function changes are measured with the hemodynamic data conversed based on the Beer-Lambertd law [5, 6]. The near-infrared spectroscopy is widely used in evaluation, such as the evaluation of brain damage among drug users who take different types of drugs.

Dresler et al., using fNIRS, studied the neurotoxic effects of drinking and the nerve recovery related to alcohol withdrawal. They divided the experimental and control groups, designed the experiments, and obtained results compatible with an increase in frontal brain activity from alcohol dependence over abstinence up to normal functioning [7]. Okada, N et al. compared the activations in the prefrontal cortex between methamphetamine-associated psychosis and schizophrenia, and obtained the similarities and differences in prefrontal cortex dysfunction between the two conditions [8]. Yamamuro et al. Took Metaphetamine abuse as the research object. The Stroop Color word task experiment was designed, and the reduced hemodynamic responses in the prefrontal cortex might reflect higher levels of importance in patients with metaphase induced psychosis were obtained [9]. Ceceli et al. demonstrate the involvement of the prefrontal cortex in emotional, cognitive, and behavioral alterations in drug addiction, with particular attention to the impaired response inhibition and salience attribution (iRISA) framework. Consistent insights from human and non-human primate studies suggest that chronic drug use leads to iRISA-based prefrontal cortex damage [10].

Human prefrontal cortex (PFC) does not only participate in the generation and control of emotions, but is deemed to be closely related to attention, cognition and motivation [11, 12]. fNIRS can detect changes in the activation of oxyhemoglobin and deoxyhemoglobin in the brain [13–16]. The NIRS detection results suggest abnormal activation of the prefrontal cortex, orbital frontal cortex and anterior cingulate gyrus among long-term methamphetamine users [17–21].

Relevant studies have found that there is a significant correlation between the neural activity in the orbitofrontal cortex and the behavioral indicators of rewards [22]. Findings on lesion studies show that orbitofrontal cortex plays a key role in cognitive flexibility [23]. Many studies on animals and humans have confirmed that damage to orbitofrontal cortex cost the cognitive flexibility of species [24, 25]. Existing studies on neuroimaging have confirmed the important role of human orbitofrontal cortex in backward learning. As orbitofrontal cortex is key to the regulation of cognitive and emotional processes (such as cognitive flexibility), regulation disorder would take place when neural changes occur in the orbitofrontal cortex [26–31]. The disorders further lead to addictive disorders. At the same time, given stimuli associated with negative results cannot change the decision-making behavior of addicts (who have high tolerance to punishment) [32, 33].

In this paper, different types of drug abusers were studied to explore the effects of abusing different drugs on the human body, so as to guide the clinical treatment for drug rehabilitation. Thirty drug abusers were selected based on the demographic scale, oral narration, and medical tests. Using the fNIRS device, the experimental paradigm was designed to obtain fNIRS data. First, the machine learning model was used to classify the different types of drug abusers. Then the statistical software was employed to analyze the OFC activations in individuals abusing different types of drugs. This study would be of great significance to the clinical treatment for drug rehabilitation.

## Methods

### Participates

Study participate criteria: 1. Meet the diagnostic criteria for disorders caused by DSM-5 psychoactive substances; 2. Patients within six months of the withdrawal period; 3. Junior high school education and above; 4. Years of Age 18–41; 5. Voluntary participation in this study and sign the informed consent form. Exclusion criteria: 1. Severe cognitive dysfunction, unable to cooperate with the completion of project-related assessment and testing; 2. Patients with severe physical diseases; 3. Severe psychotic symptoms; 4. Have other mental activities Substance abuse (except nicotine). The study was conducted in accordance with the declaration of Helsinki and was approved by the Ethics Committee of Shanghai University (Approval No. ECSHU2020–071).

### Drug users description

According to the demographic scale made in the early stage, the most important items of 30 people (All male), such as drug use type, drug history, average drug dosage and drug use frequency, were accurately inquired and counted.

Methamphetamine easily causes intense excitement, which is difficult to eradicate after addiction.

Heroin is a psychoanaesthetic drug. Once a person becomes addicted, their physiological reaction is intense, and they have a compulsion to seek medication.

“Mixed drug abusers” refers to drug users who attempt to mix two or three drugs at a time. The main types of drugs used are: LSD, Flunitrazepam, N_2_O, Pethidine hydrochloride, MDMA, Cannabis, ketamine, etc. Table 1 is the personal information of the selected 30 subjects.

**Table 1:** Personal information of drug addicts

N	30
Sex(M/F)	Male
Age range (Year)	19–41
Years of education	10±2.72
Years of drug abuse	8.2 ± 4.74
Drug abuse per week:	Most people 3–5 times a week.
withdrawals	1.4 ± 0.98
Reasons for taking drugs	decompression needs ; sex ; emotion; curiosity; emotional frustration;

### NIRS technology equipment introduction

**Fig. 1:**
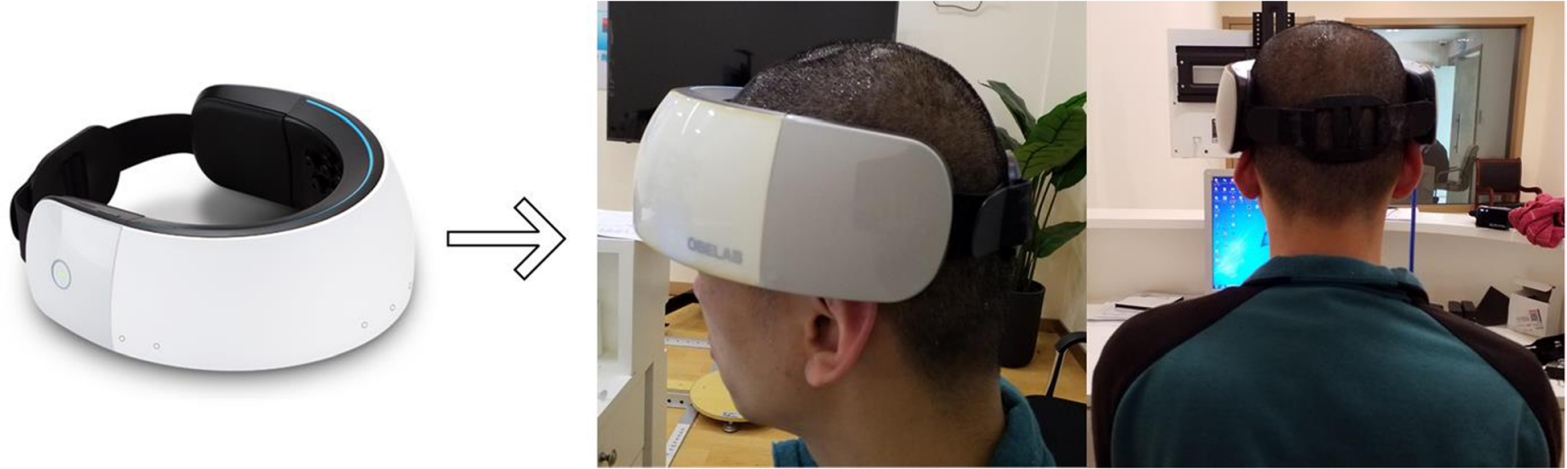
NIRSIT wearing method in the experiment

This paper uses a high-density NIRS device (NIRSIT; OBELAB, Seoul, Korea), and its specific hardware parameters and wearing methods are as follows: the specific hardware parameters are shown in Table 2. Figure 1 NIRSIT wearing method in the experiment.

**Table 2:** NIRSIT specific hardware parameters

NIRSIT	OBELAB, Seoul, Korea
Light source type	dual-wavelength VCSEL laser
Light source technology:	CW
Wavelength	780 nm, 850 nm
Number of light sources	24
Number of detectors	32
SD distance	1.5 cm, 2.12 cm, 3.0 cm, 3.35 cm
Number of channels	204
Detection distance	0.2 cm, 0.6 cm, 1.0 cm, 1.4 cm, 1.8 cm
Spatial resolution	4x4mm^2^
Sampling frequency	8.13 Hz

### NIRS channel and functional area division

**Fig. 2:**
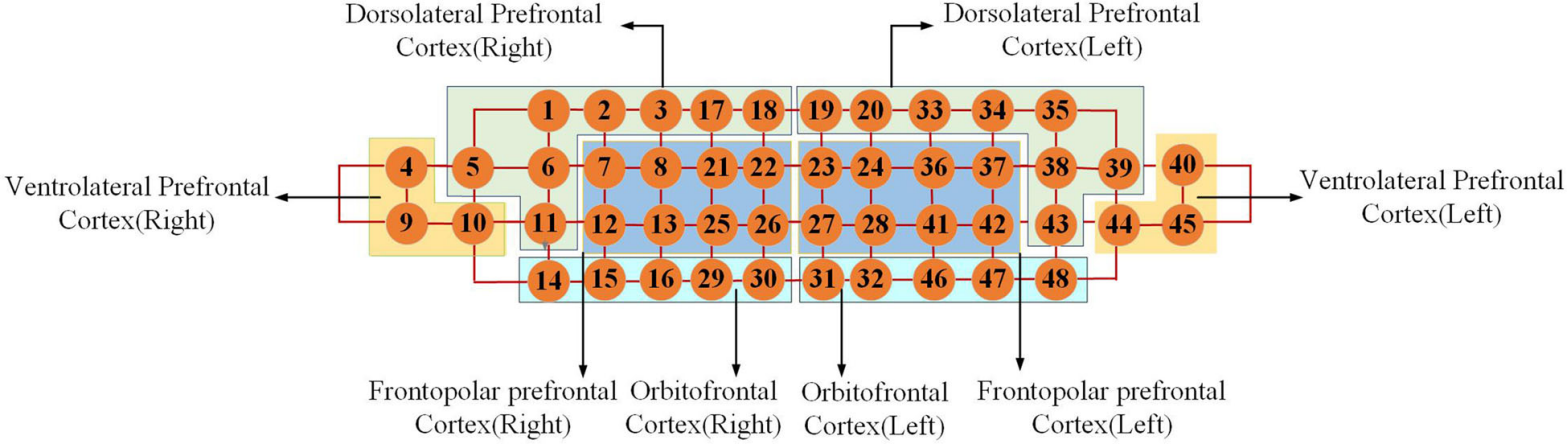
NIRS channel and functional area division

The four advanced functional areas detected by the forehead fNIRS device are divided into the dorsolateral prefrontal cortex, the ventrolateral prefrontal cortex, frontopolar prefrontal cortex, and the orbital frontal cortex. The specific channel distribution: the right dorsolateral prefrontal cortex is 1,2,3,5,6,11,17,18 channels. There were 19, 20, 33, 34, 35, 38, 39 and 43 channels in the left dorsolateral prefrontal lobe. There are 4,9,10,40,44,45 channels in ventrolateral prefrontal cortex of left and right hemispheres. There are 14, 15, 16, 29, 30, 31, 32, 46, 47, 48 channels in the left and right orbital frontal cortex. Frontopolar prefrontal cortex is 7,8,12,13,21,22,23,24,25,26,27,28,36,37,41,42 channels. Fig. 2 NIRS channel and functional area division.

### Near-infrared imaging theory

When light passes through a uniform, non-scattering medium, only the absorption effect of the medium on the photons is considered. According to the Beer–Lambert law [34–37], the attenuation of light intensity is expressed as follows:

$$ OD=\log \frac{I}{I_0}=-\upvarepsilon \left(\lambda \right) cd\kern0.1em \log \kern0.3em \mathrm{e} $$

where *I*_0_ is the incident light intensity, *I* is the incident light intensity, *ε*(*λ*) is the extinction coefficient of the substance at a wavelength of *λ*, determined by the absorbing medium and the wavelength of the light, *c* is the medium concentration, and *d* is the thickness of the medium. The absorption coefficient *μ*_*a*_ is defined as follows:

$$ {\mu}_a=\varepsilon \left(\lambda \right)c $$

The total absorption coefficient of the medium can be expressed as a linear superposition of the absorption coefficients of each medium:

$$ {\mu}_a\left(\lambda \right)=\sum \limits_i^N{\varepsilon}_i\left(\lambda \right){c}_i $$

The optical density is the product of the medium thickness *d* and the total absorption coefficient *μ*_*a*_ [38, 39]:

$$ OD=\mathit{\log}\frac{I}{I_0}=d\sum \limits_i^N{\varepsilon}_i\left(\lambda \right){c}_i $$

The actual biological tissue is very complex and is a strong light scatterer, and light undergoes multiple scatterings during its output before it can be detected. The attenuation of light in tissues includes absorption and scattering, and to capture the effect of scattering on light loss, Deply et al. proposed a modified Beer–Lambert law [40], expressed as follows:

$$ OD=\log \frac{I}{I_0}= DPF\left(\lambda \right)\cdot dc\upvarepsilon +G $$

where *G* denotes the light loss due to scattering and other boundary losses. *DPF* is the differential path factor, whose value is the ratio between the actual optical path length traveled by light in the tissue and *d*. The DPF values in different tissues can be obtained from the literature [41].

### Measurement of changes in hemodynamic parameters

To detect changes in hemodynamic parameters using NIRS, a reference state is usually selected in near-infrared measurements to detect relative changes in the concentration of absorbing chromophores *Δ*_*c*_, based on the modified Beer–Lambert law [42, 43]:

$$ \varDelta OD=\mathit{\log}\frac{I}{I_0}= DPF\left(\lambda \right)\cdotp d\varDelta c\varepsilon $$

When detecting relative changes in *HbO*2 and *HHb* concentrations:

$$ \varDelta O{D}^{\lambda }=\left({\varepsilon}_{Hbo}^{\lambda}\varDelta \left[ Hb{O}_2\right]+{\varepsilon}_{HHb}^{\lambda}\varDelta \left[ HHb\right]\right) DPF\left(\lambda \right)\cdotp d $$

where *Δ*[*HbO*_2_] and *Δ*[*HHb*] are the variations in *HbO*_2_and *HHb* concentrations, the selected incident near-infrared wavelengths *λ*_1_ and *λ*_2_, brought into the above equation have [44, 45].

$$ \varDelta O{D}^{\lambda_1}=\left({\varepsilon}_{Hbo}^{\lambda_1}\varDelta \left[ Hb{O}_2\right]+{\varepsilon}_{HHb}^{\lambda_1}\varDelta \left[ HHb\right]\right) DPF\left({\lambda}_1\right)\cdotp d $$

$$ \varDelta O{D}^{\lambda_2}=\left({\varepsilon}_{Hbo}^{\lambda_2}\varDelta \left[ Hb{O}_2\right]+{\varepsilon}_{HHb}^{\lambda_2}\varDelta \left[ HHb\right]\right) DPF\left({\lambda}_2\right)\cdotp d $$

When the value of *DPF* is known, solving the above equation for the system of equations yields the change in *HbO*_2_ concentration *Δ*[*HbO*_2_] and the change in *HHb* concentration *Δ*[*HHb*], expressed as follows:

$$ \varDelta \left[ Hb{O}_2\right]=\frac{\varepsilon_{HHb}^{\lambda_2}\frac{\varDelta O{D}^{\lambda_1}}{DPF\left({\lambda}_1\right)}-{\varepsilon}_{HHb}^{\lambda_1}\frac{\varDelta O{D}^{\lambda_2}}{DPF\left({\lambda}_2\right)}}{d\left({\varepsilon}_{HHb}^{\lambda_2}{\varepsilon}_{Hb{O}_2}^{\lambda_1}-{\varepsilon}_{HHb}^{\lambda_1}{\varepsilon}_{Hb{O}_2}^{\lambda_2}\right)} $$

$$ \varDelta \left[ HHb\right]=\frac{\varepsilon_{Hb{O}_2}^{\lambda_2}\frac{\varDelta O{D}^{\lambda_1}}{DPF\left({\lambda}_1\right)}-{\varepsilon}_{Hb{O}_2}^{\lambda_1}\frac{\varDelta O{D}^{\lambda_2}}{DPF\left({\lambda}_2\right)}}{d\left({\varepsilon}_{HHb}^{\lambda_2}{\varepsilon}_{Hb{O}_2}^{\lambda_1}-{\varepsilon}_{HHb}^{\lambda_1}{\varepsilon}_{Hb{O}_2}^{\lambda_2}\right)} $$

### Experiment design and data acquisition

We used E-prime software package (Psychology Software Tools, Pittsburgh, PA) to write the experimental paradigm, with each map numbered. A complete experimental paradigm consists of the following three stages.

The first stage of the experimental paradigm, 10 min in total, during which the subjects need to close their eyes for 5 min and then open their eyes for 5 min.

**Fig. 3:**
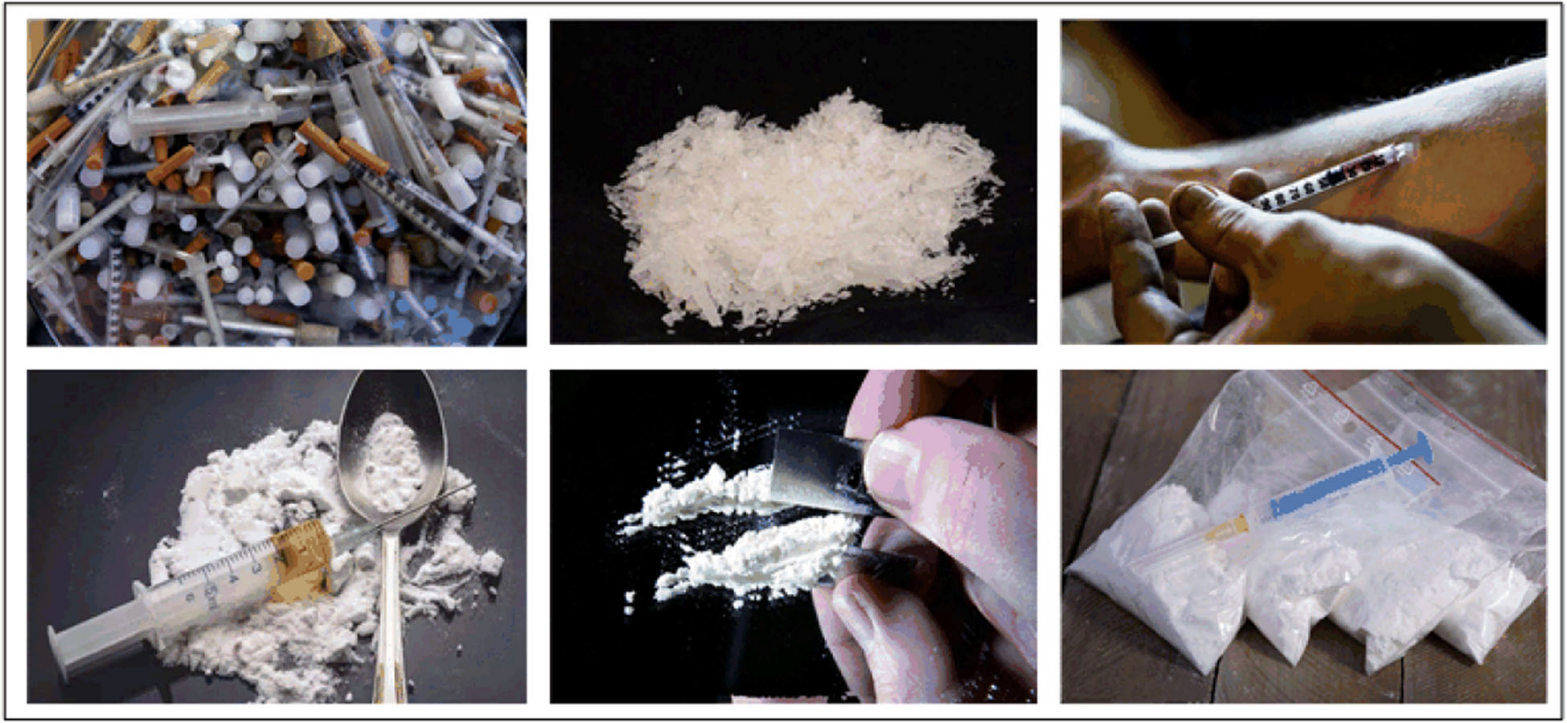
Examples of drug abuse-related maps in the experimental paradigm

The second stage, it lasts 6 min and is divided into drug maps and neutral maps. Among them, each block lasts 10 s. There are a total of 16 maps, and the display time of each map is 0.6 s. At the beginning, the first four maps are displayed randomly, during which there are two drug maps. After displaying the first four maps, the remaining 12 neutral maps are displayed randomly. After a block ends, there will be a 4-s interval map with a white background and a black cross. Figure 3, the examples of drug abuse-related maps used in the experimental paradigm.

**Fig. 4:**
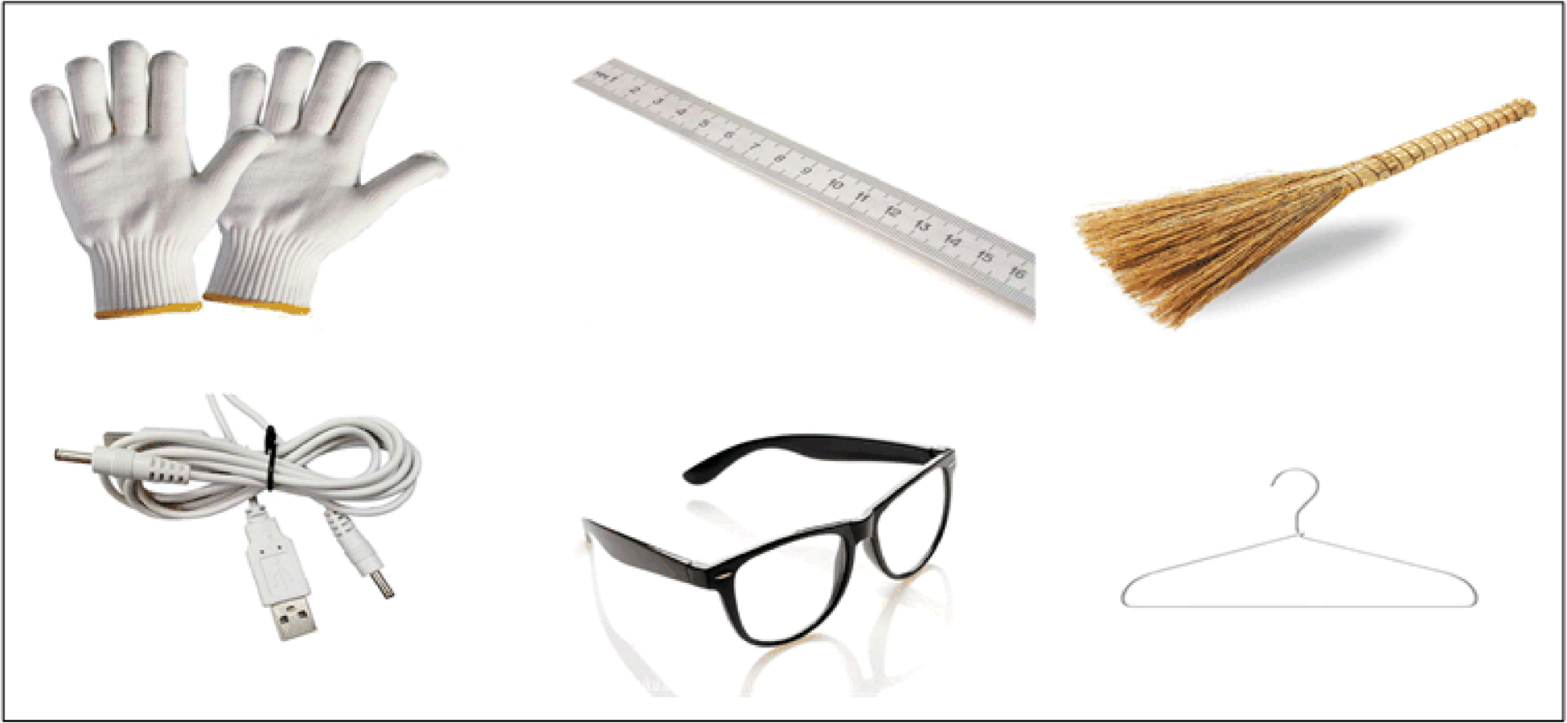
Examples of neutral maps in the experimental paradigm

**Fig. 5:**
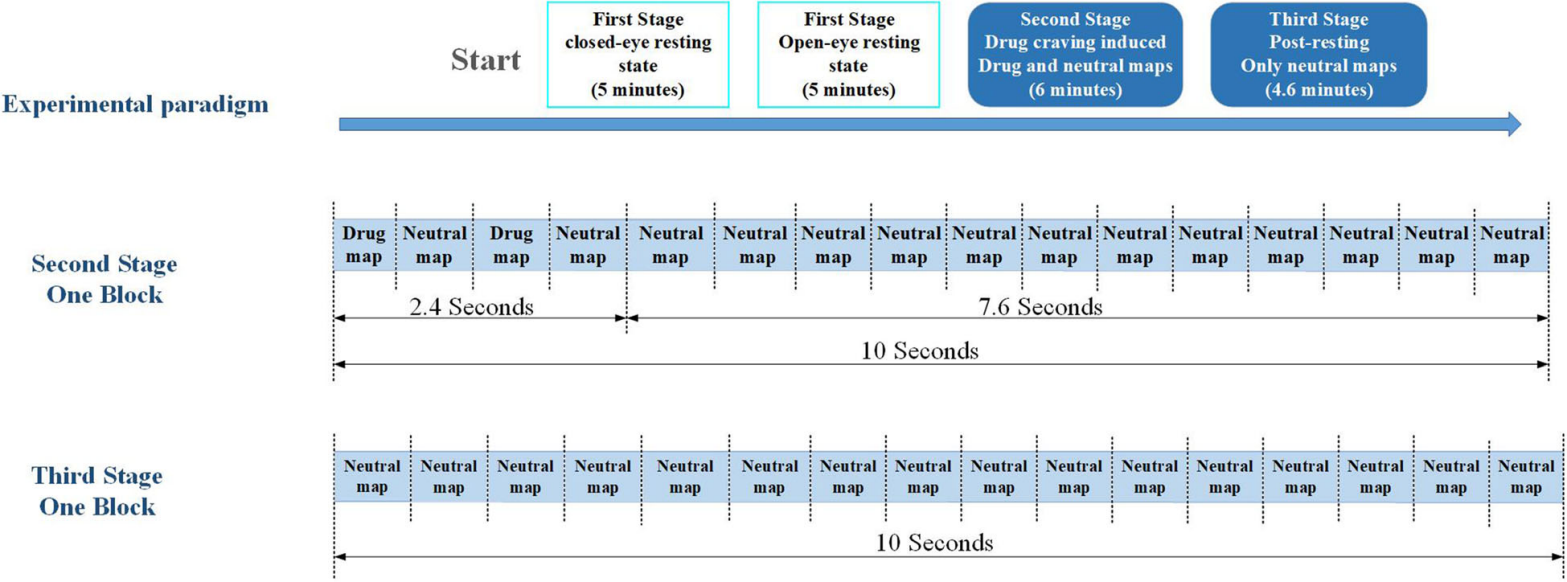
The whole process of experimental paradigm

The third stage, it lasts a total of 4.6 min, during which the maps are all neutral, with each block lasting 10 s. There are 16 maps in total, with a display speed of 0.6 s. There will be a 4-s interval between each block. Figure 4, the examples of neutral maps used in the experimental paradigm. Figure 5, The whole process of experimental paradigm.

### Linear discriminant analysis algorithm principle

Linear discriminant analysis (LDA) is a supervised pattern recognition method. The LDA classifier reduces the dimensions of the data, reduces complex features into low-dimensional features through projection, and searches for specific classification surfaces to maximize the discrimination between the two types of task classification in order to realize feature classification [46].

LDA is the most traditional linear classifier; there are k linear functions for a k-classification problem.

$$ {y}_K(x)={w}_k^Tx+{w}_{k_0} $$

When *y*_*k*_ > *y*_*j*_ for all j, then x belongs to class k. When *k* = 2, it becomes a binary problem.

### Support vector machine algorithm principle

Support vector machine (SVM): For nonlinear feature input, we design mapping rules to map nonlinear features to high-dimensional space and ensure that the features are distributed linearly in high-dimensional space. Then, we can easily construct the optimal classification hyperplane in high-dimensional space based on the structural risk minimization criterion so that this optimal classification hyperplane can correctly separate as many of the two types of samples on the one hand and maximize the classification interval between the two types on the other [47].

The sample set is designated as (*x*_*i*_, *y*_*i*_), *i* = 1, 2, ⋯, *l*, *x*_*i*_ ∈ *R*^*d*^, where *y*_*i*_ ∈ {1, −1} is the category number. The linear separable and linear non-separable cases are explored jointly, and the relaxation variable is introduced *ξ*_*i*_ ≥ 0, where *ξ*_*i*_ = 0 represents linear separability, and *ξ*_*i*_ > 0 represents nonlinear separability. If the classification surface equation is *w* · *x* + *b* = 0 (*w* is the weight vector, and *b* is the offset), the classification interval is equal to $\frac{2}{\left\Vert w\right\Vert }$. Maximizing the classification interval is equivalent to minimizing ‖*w*‖ or (‖*w*‖^2^). To make the classification surface classify all samples correctly as much as possible, it is necessary to meet the following constraints:

$$ {y}_i\left[\left(w\cdotp {x}_i\right)+b\right]-1+{\xi}_i\ge 0,i=1,2,\cdots, l $$

Therefore, if the constraint formula is satisfied, the (‖*w*‖^2^) minimum classification surface can be the optimal classification surface.

### Machine learning algorithm classification

**Fig. 6:**
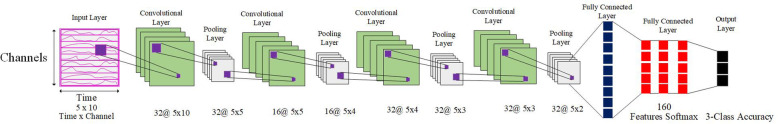
Structure of convolutional neural network

In this paper, we propose and design a neural network algorithm model that extracts the hidden features in the 0.625-s fNIRS data after drug map stimulation by convolution to define the type of drugs of abuse. In this paper, a convolutional neural network (CNN) model is designed. It is used to implement the classification of people who abuse different type of addictive drugs. The Structure of CNN used in this paper is shown in Fig. 6.

### Data preprocessing

Butterworth filter, the frequency response curve in the passband has a relatively flat and undulating character, and gradually decreases to zero at the edge of the stopband. In this paper, a Butterworth filter based on infinite impulse response is selected to band-pass filter the acquired Near infrared signals for the purpose of physiological artifact removal. The expression for the n-order Butterworth filter is as follows:

$$ {\left|\boldsymbol{H}\left(\boldsymbol{f}\right)\right|}^{\mathbf{2}}=\frac{\mathbf{1}}{\mathbf{1}+{\left(\frac{\boldsymbol{f}}{{\boldsymbol{f}}_{\boldsymbol{c}}}\right)}^{\mathbf{2}\boldsymbol{n}}}=\frac{\mathbf{1}}{\mathbf{1}+{\boldsymbol{\upepsilon}}^{\mathbf{2}}{\left(\frac{\boldsymbol{f}}{{\boldsymbol{f}}_{\boldsymbol{p}}}\right)}^{\mathbf{2}\boldsymbol{n}}} $$

Where n is the order, ***f***_***c***_ is the cutoff frequency, and ***f***_***p***_ is the passband edge frequency.

In this paper, n is 6 and the frequency band range is 0.01 Hz to 3 Hz. This band range can remove the interference of heartbeat respiration and slow drift to the raw data, and also can maximize the preservation of hemodynamic characteristics.

The formula for each structure in the CNN network is as follows:

### Convolutional layer

Convolutional layers are the core of convolutional neural networks [48]. The calculation form is as follows:

$$ {x}_j^l=f\left(\sum \limits_{i\in {M}_j}{x}_i^{l-1}\cdotp {k}_{ij}^l+{b}_j^l\right) $$

${x}_j^l$ is the *j*th feature of the layer *l*.${k}_{ij}^l$ is the *j*th feature of the layer *l* and the *i*th feature of the layer *l* − 1. ${b}_j^l$ is a bias parameter, *f*(•) is the activation function.

### Pooling layer

The pooling layer sub-samples the input features according to specific rules in order to make the network robust to small changes in previously learned features [49]. The calculation form is as follows:

$$ {x}_j^l=f\left({\upbeta}_1^l\backslash down\left({x}_j^{l-1}\right)+{b}_j^l\right) $$

${x}_j^l$ is the *j*th feature of the layer *l*.${\beta}_1^l$ is the Subsampling coefficient.${b}_j^l$ is the bias parameter, *down*(•) is a sub-sampling function, *f*(•) is the activation function.

### Normalization of data

Batch standardization layer: in training convolutional neural network, the input data are usually whitened, which can speed up the training speed.

Set the data value *input* = {*x*_1_…*x*_*m*_} of the input data block, the parameters to be learned are *γ* and *β*, first calculate the average value of each data block:

$$ {\mu}_B=\frac{1}{m}\sum \limits_{i=1}^m{x}_i $$

Calculate the variance of each data block:

$$ {\sigma}_B^2=\frac{1}{m}\sum \limits_{i=1}^m{\left({x}_i-{\mu}_B\right)}^2 $$

Normalize each set of data:

$$ {\hat{x}}_i=\frac{x_i-{\mu}_B}{\sqrt{\sigma_B^2+\varepsilon }} $$

Using the parameters that need to be learned in the network and linear transformation:

$$ {y}_i=\gamma {\hat{x}}_i+\beta \equiv B{N}_{\gamma \beta}\left({x}_i\right) $$

### Activation function

In this paper, the activation function uses a modified linear unit (ReLU), and the corresponding calculation formula is as follows:

$$ \mathit{\operatorname{Re}}L\mathrm{U}=\left\{\begin{array}{c}x,x\ge 0\\ {}x,x<0\end{array}\right.=\mathit{\max}\left(0,x\right). $$

The results show that the derivation of the activation function is simple, and the output of some neurons is 0, which realizes the sparsity of the network, reduces the interdependence of parameters [50].

### Full connection layer

Each feature must be converted to one-dimensional before it can be used as the input of the fully connected layer [51]. The calculation as follows:

$$ {h}_{w,b}(x)=\uptheta \left({w}^{\mathrm{T}}x+b\right) $$

*h*_*w*, *b*_(*x*) is the output value of the neuron. *x* is the input feature vector of the neuron. *w* is the weight. *b* is the bias parameter. θ(∙) is the activation function; The first fully connected layer in this paper uses the ReLU activation function.

### Softmax layer

In CNN, if the final output result is single-label multi-classification, the softmax function is usually used to normalize and map to the probability value, and the Softmax calculation formula is as follows:

$$ {z}_i=\mathrm{Softmax}\left({o}_i\right)=\frac{\exp \left({o}_i\right)}{\sum_c\exp \left({o}_c\right)} $$

*o*_*i*_ is the value of the output neuron corresponding to the Ith category.

## Results

### Accuracy comparison of LDA, SVM, and CNN

LDA and SVM use the original data as the training set and test set. The classification model selects 21 data points as the training set and 9 as the test set and cross validates the model. Finally, we obtain their own 3-class accuracy. The classification accuracy of LDA is stable between 45 and 58%. The classification accuracy of SVM is stable between 60 and 69%.

The feature extraction method and classification accuracy of the CNN model are as follows:

The data feature 16 channels and 56 trials for each subject. The data fragment is 0.625 s after the drug picture appears. The CNN network includes 24 subjects’ training data, 3 subjects’ validation data and 3 subjects’ testing data.

**Fig. 7:**
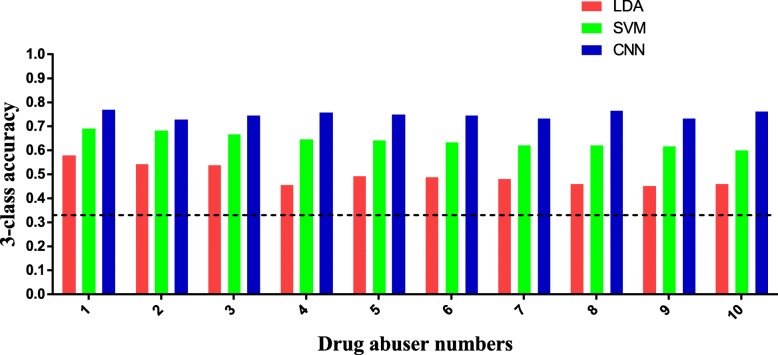
Comparison of 3-class accuracy between LDA, SVM, and CNN

The CNN model shows a stable decreasing trend in loss and convergence after 1200 epochs, which proves that the network structure is stable. After several experiments, the optimal model basically appears between 1000 and 1300 epochs. At this time, the 3-class accuracy of different drug abusers is between 70 and 80%. The accuracy rates tested using LDA, SVM, and CNN algorithms, respectively, are given in the table. A comparison between the three is shown in the figure. Table 3 Three-class accuracy statistics. Figure 7 Comparison of 3-class accuracy between LDA, SVM and CNN.

**Table 3:** 3-class accuracy statistics

	1	2	3	4	5	6	7	8	9	10
LDA	58.24%	54.29%	53.83%	45.66%	49.52%	48.90%	47.96%	46.19%	45.24%	45.91%
SVM	69.39%	68.57%	66.90%	64.83%	64.29%	63.27%	62.38%	62.09%	61.81%	60%
CNN	77.04%	72.96%	74.74%	75.77%	75.26%	74.59%	73.47%	76.79%	73.47%	76.28%

### Analysis methods of drug abusers

Statistical analyses were performed using GraphPad Prism 6.5 software (GraphPad Software Inc., San Diego, USA). The independent sample *t*-test and the non-parametric Mann–Whitney U test were used in the study. The relevant laws of brain activation of people who abuse different drugs were obtained. Especially in the OFC functional area of the brain, people who abuse different drugs obtain significantly different results.

### Right-OFC activated for people who abuse different drugs

To begin with, five channels in both the left and right hemispheres of the brain of 30 addicts who take heroin, methamphetamine and mixed drugs were chosen to get the near-infrared data induced by drug pictures. Then the mean of OFC data of 30 subjects was calculated. The data of each type of drug addicts was saved in one column and processed with GraphPad Prism6.5 software.

For Heroin VS Methamphetamine in right OFC, the independent sample T test results were t = 1.020, df = 98, *P* = 0.312, F = 31.90, DFn = 49, Dfd = 49, *P* < 0.0001; and for Mixed drug VS Methamphetamine, the results were t = 0.7409, df = 98, *P* = 0.4605, F = 1.048, DFn = 49, Dfd = 49, *P* = 0.8707.

**Fig. 8:**
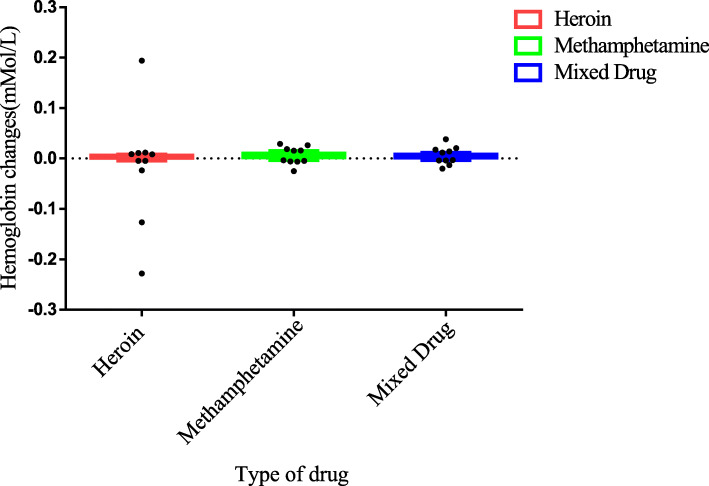
Right-OFC and Left-OFC activation differences among the three types of drug abusers

For Heroin VS Mixed drug, the results were t = 0.8400, df = 98, *P* = 0.4030, F = 33.42, DFn = 49, Dfd = 49, *P* < 0.0001. This is caused by heterogeneity variance. For Heroin VS Methamphetamine, the results of the non-parametric Mann Whitney test were Exact, Two-tailed, *P* = 0.0048 < 0.05 (significant difference); for Methamphetamine VS Mixed drug, the results were Exact, Two-tailed, *P* = 0.2215 (insignificant difference); and for Heroin VS Mixed drug, the results were Exact, Two-tailed, *P* = 0.0852 (insignificant difference). As for the activation of right OFC by three types of drugs (in mMol/L), the HbO2 of heroin addicts was − 0.0007433 ± 0.04683, methamphetamine addicts 0.006117 ± 0.008292, and mixed addicts 0.004902 ± 0.00810. The activation of right OFC is highest among methamphetamine addicts, followed by mixed types and heroin. The result is the same for left- OFC. Figure 8, Right-OFC and Left-OFC activation differences among the three types of drug abusers.

### Activation of the left and right hemispheres of the brain in drug addicts

**Fig. 9:**
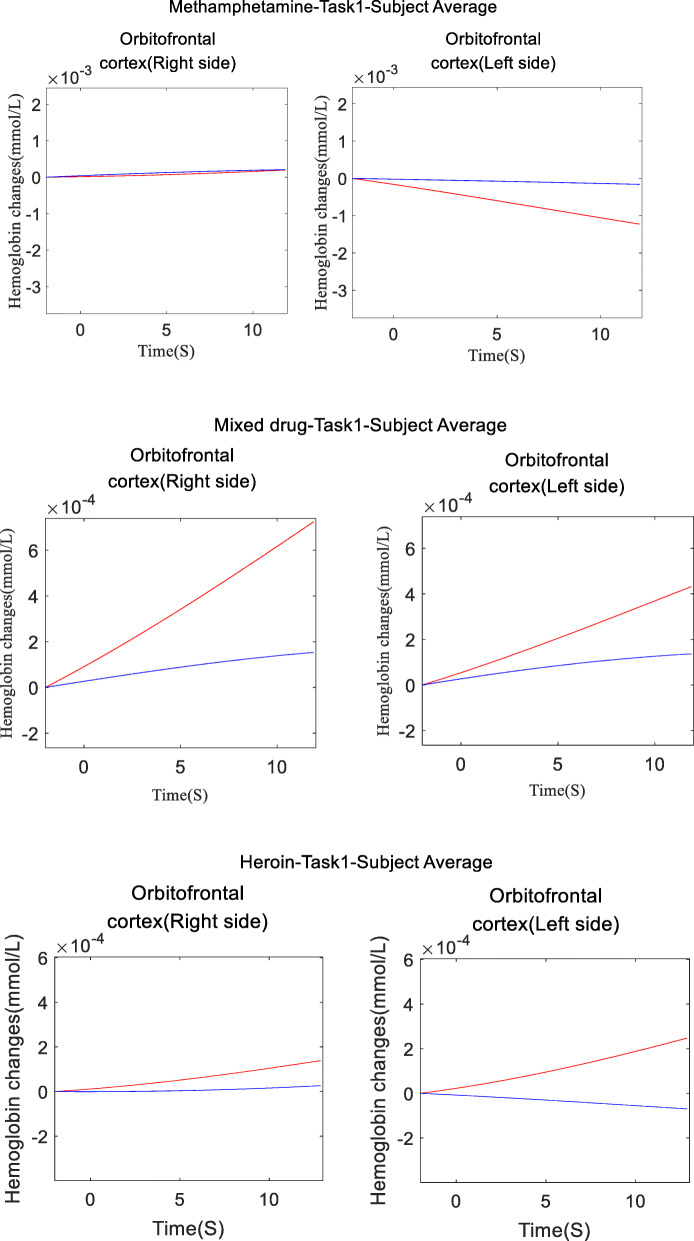
time series of hemoglobin concentration

For methamphetamine, mixed drugs, and heroin addicts, the left and right hemispheres of the brain are activated in OFC. The average values of the oxygenated hemoglobin concentration and deoxygenated hemoglobin concentration of the three types of subjects within 0–10 s are shown in Fig. 9. There is a difference in activation between the left and right hemispheres of the brain.

### Abuse of different drugs differs in OFC activation

**Fig. 10:**
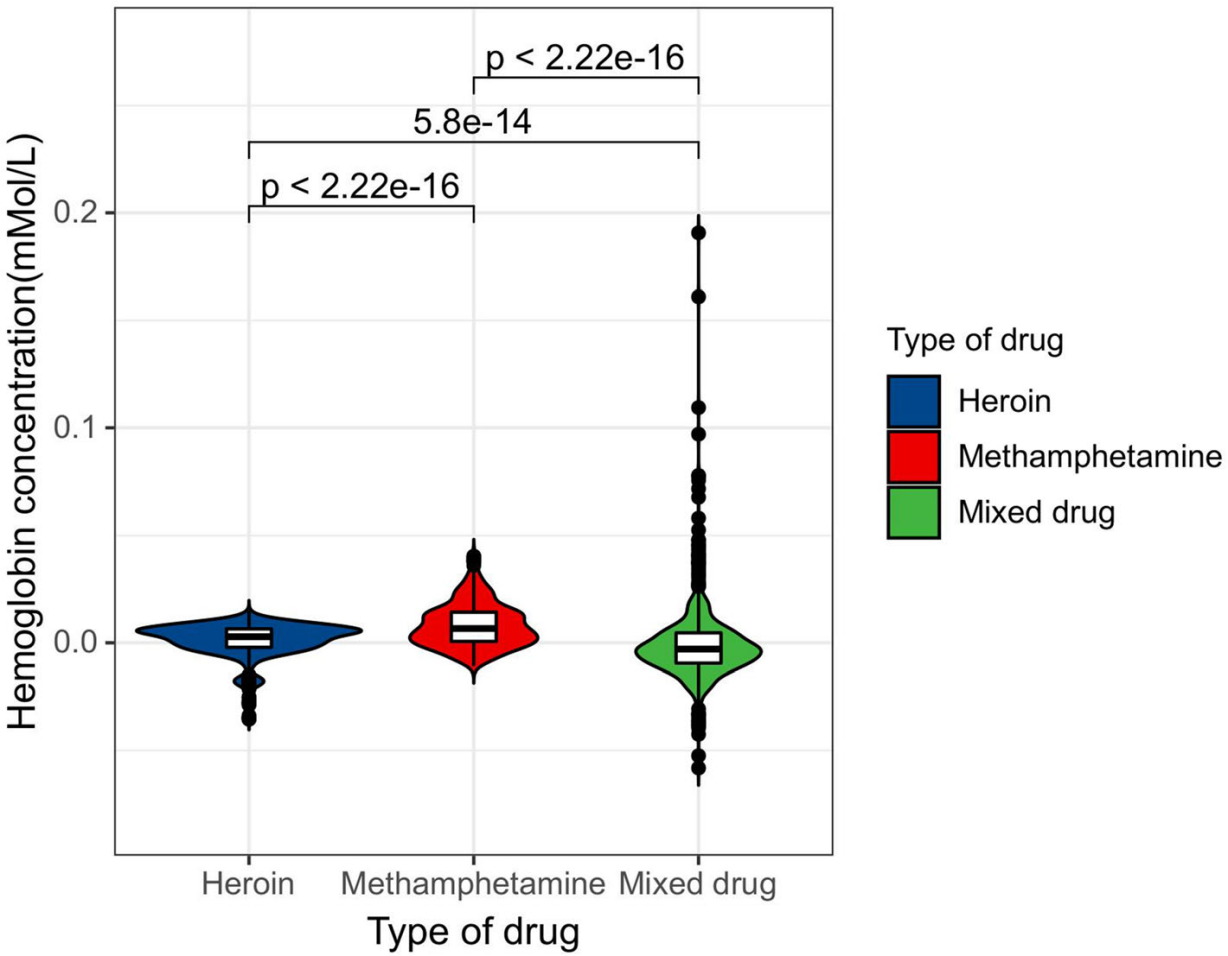
OFC activation differences among the three types of drug abusers

The results of activation of the three types of personnel on OFC were analyzed using a non-parametric Kruskal–Wallis test. The number of tested groups was three, and the total number of values was 300. The Kruskal–Wallis test statistic was 8.355 (*P* = 0.0153). There were significant differences among the three types of personnel. Activation of the right OFC was the highest among methamphetamine addicts, followed by mixed types, and lastly, heroin addicts. Figure 10 shows differences in OFC activation among the three types of drug abusers.

### Differences in prefrontal cortex activation across drug addicts

**Fig. 11:**
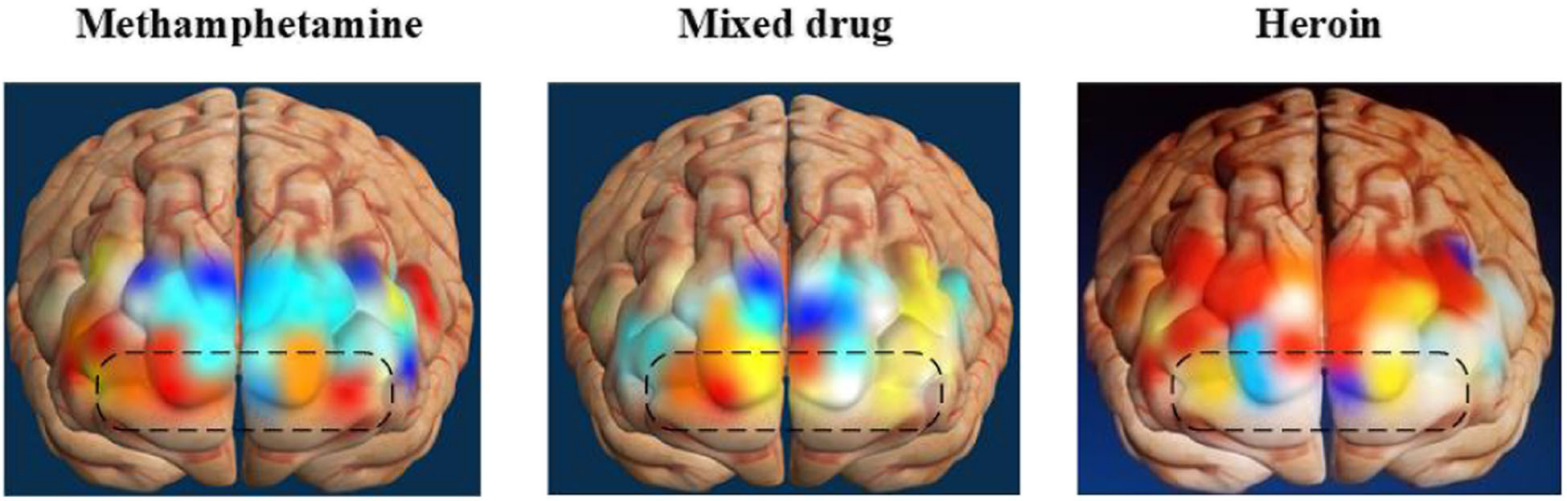
Taking different type of drug addicts brain OFC activation

From the prefrontal cortex activation results in Fig. 11, we can see the differences in activation of the OFC cortex in the black box. The darker the red color, the higher the degree of activation. Methamphetamine abusers had the highest average blood oxygen levels in the OFC, followed by mixed drug abusers, and heroin abusers had the lowest.

## Discussion

This study first uses the LDA, SVM and machine-learning algorithm to classify the addicts of heroin, methamphetamine and mixed drugs. Analysis on the brain activation and OFC activation is made to three types of drug addicts, which is of clinical significance.

Based on the analysis of addicts who take different types of drugs, the research finds that the activation of right OFC is highest among methamphetamine addicts, followed by mixed types and heroin. The result is the same for left OFC, but with significant difference in the methamphetamine group. The findings show the difference in the impact on human bodies among addicts who take heroin, methamphetamine and mixed drugs.

The final results obtained demonstrate that the abuse of different drugs produces differences in OFC activation. OFC activation changes after the addicts use drugs. The results are consistent with those arrived at by other scholars. Some researchers only proved the difference in OFC between drug addicts and healthy people. No research has been made to study the differences in prefrontal cortex activation caused by different drugs.

The four characteristics of addiction: impaired cognitive and motivational functions, increased craving for addictive cues, preference for rewards while ignoring risks, and impulse control disorder [52–54]. Another typical feature of addiction is that the individuals tend to pursue rewards for addictive behaviors while ignoring the risks in their cognitive decision-making [55, 56]. Heroin addicts showed higher impulsivity in the Go/Nogo experimental tasks, and the activation in their cognitive control loop was weakened [57]. Long-term abuse of addictive drugs not only harms the physical and mental health of addicts, but also impairs their social functions. Meanwhile, it also causes serious damages to the cognitive processes of addicts, such as focus and concentration, verbal memory, and executive capacity [58, 59]. At present, a large amount of evidence shows that the brain areas related to the cognitive and emotional processing of drug addicts are damaged to varying degrees by the abuse of addictive drugs [60].

Changes in OFC activation cause changes in cognition, control, and emotions. Differences in OFC activation among addicts who take different types of drugs lead to the conclusions that: Methamphetamine has the most serious impact on the human body; There are differences in the harm of different drugs; Customized drug rehabilitation schemes should be developed; Cognitive and control training is conducive to drug rehabilitation.

The limitation of this research lies in the limited amounts of subjects, which could make the findings less convincing. In future experiments, more subjects should be included and comparison between the outcomes of customized rehabilitation and those of traditional rehabilitation should be made.

The experiment proves that different drugs have varied impacts on the addicts, which requires customized drug rehabilitation schemes.

## Conclusion

This paper points out that different drugs affect the brain differently. The brain activation pattern of heroin, methamphetamine and mixed drugs is arrived at, filling up the blank on the study about different types of drugs. The findings are valuable for future clinical diagnosis and customized rehabilitation.

This research finds differences in OFC activation among addicts who take different types of drugs, and arrives at the conclusion that the right OFC activation of methamphetamine abusers is the highest, followed by that of mixed drug abusers and heroin abusers. The conclusion with left OFC activation is the same.

Existing research mostly focuses on moods, cognition and attention when studying drug rehabilitation. This paper introduces the fNIRS device to obtain the physiological data, and performed intelligent algorithm analysis and statistical analysis. The findings are consistent with existing conclusions that drugs have impact on brain functions. This research is innovative in that it makes classified study on the addicts who use different types of drugs. In theory, this paper enriches the empirical discussion on the impact of different types of drugs on human brains; and in practice, it provides guidance on customized drug rehabilitation.

## Data Availability

All data used in this article are from Qingdong Drug Rehabilitation Center, Shanghai, China. The data is currently in a confidentiality agreement and cannot be disclosed.

## Abbreviations

fNIRS: functional near-infrared spectroscopy
LDA: Linear discriminant analysis
SVM: Support vector machine
CNN: Convolutional Neural Network
PFC: Prefrontal Cortex
FPC: Frontpolar prefrontal cortex
OFC: Orbitofrontal Cortex
VLPFC: Ventrolateral prefrontal Cortex
DLPFC: Dorsolateral prefrontal cortex
EEG: electroencephalography
HbO2: oxyhemoglobin
Hbb: deoxyhemoglobin
MBLL: Modified lambert-beer law
NIRS: NIRSIT
DSM-5: Diagnostic and Statistical Manual of Mental Disorders Five Edition
LSD: lysergic acid diethylamide
MDMA: 3,4-Methylenedioxymethamphetamine
N_2_O: Nitrous oxide
PFC: Prefrontal cortex
iRISA: impaired response inhibition and salience attribution
